# Heterogeneous *MYCN* amplification in neuroblastoma: a SIOP Europe Neuroblastoma Study

**DOI:** 10.1038/s41416-018-0098-6

**Published:** 2018-05-14

**Authors:** Ana P. Berbegall, Dominik Bogen, Ulrike Pötschger, Klaus Beiske, Nick Bown, Valérie Combaret, Raffaella Defferrari, Marta Jeison, Katia Mazzocco, Luigi Varesio, Ales Vicha, Shifra Ash, Victoria Castel, Carole Coze, Ruth Ladenstein, Cormac Owens, Vassilios Papadakis, Ellen Ruud, Gabriele Amann, Angela R. Sementa, Samuel Navarro, Peter F. Ambros, Rosa Noguera, Inge M. Ambros

**Affiliations:** 1 Department of Pathology, Medical School, University of Valencia/INCLIVA Biomedical Research Institute, 46010 Valencia, Spain; 2 Ciberonc, Madrid, Spain; 3 Department of Tumour Biology CCRI, Children’s Cancer Research Institute, St. Anna Kinderkrebsforschung, 1090 Vienna, Austria; 4 S2IRP: Studies and Statistics for Integrated Research and Projects CCRI, Children’s Cancer Research Institute, St. Anna Kinderkrebsforschung, 1090 Vienna, Austria; 5 Institute of Clinical Medicine, Faculty of Medicine, University of Oslo and Department of Pathology, Norwegian Radium Hospital, Oslo University Hospital, 0372 Oslo, Norway; 6 Northern Genetics Service, The Newcastle upon Tyne Hospitals NHS Foundation Trust, Institute of Genetic Medicine, Central Parkway, Newcastle upon Tyne, NE1 3BZ UK; 7 Centre Léon Bérard, Laboratoire de Recherche Translationnelle, 28 rue Laennec, Lyon, 69008 France; 8 Department of Pathology, Gaslini Institute, Largo G. Gaslini 5, 16147 Genoa, Italy; 9 Cancer Cytogenetic and Molecular Cytogenetic Laboratory, Schneider Children’s Medical Center of Israel, 49202 Petach Tikva, Israel; 10 Laboratory of Molecular Biology, Gaslini Institute, Largo G. Gaslini 5, 16147 Genoa, Italy; 11 Department of Pediatric Hematology and Oncology, Charles University in Prague, Second Faculty of Medicine and University Hospital Motol, 15006 Prague, Czech Republic; 12 Department of Paediatric Haematology-Oncology, Schneider Children’s Medical Center of Israel, 49202 Petach Tikva, Israel; 13 Pediatric Oncology Unit, Hospital Universitari i Politècnic La Fe, 46026 Valencia, Spain; 14 Department of Paediatric Haematology-Oncology, Aix-Marseille University and APHM, Hôpital d’ Enfants de La Timone, 13385 Marseille, France; 15 St Anna Children’s Hospital and Department of Paediatrics of the Medical University, 1090 Vienna, Austria; 16 Our Lady’s Children’s Hospital, Crumlin, Dublin, D12 N512 Ireland; 17 Department of Paediatric Haematology-Oncology, Agia Sofia Children’s Hospital Athens, 11528 Athens, Greece; 18 Department of Paediatric Medicine, Rikshospitalet, Oslo University Hospital, 0372 Oslo, Norway; 19 Institute of Clinical Pathology, Medical University Vienna, Vienna, Austria; 20 Department of Paediatrics, Medical University Vienna, Vienna, Austria

**Keywords:** Paediatric cancer, Paediatric cancer, Cancer genomics

## Abstract

**Background:**

In neuroblastoma (NB), the most powerful prognostic marker, the *MYCN* amplification (MNA), occasionally shows intratumoural heterogeneity (ITH), i.e. coexistence of *MYCN*-amplified and non-*MYCN*-amplified tumour cell clones, called heterogeneous MNA (hetMNA). Prognostication and therapy allocation are still unsolved issues.

**Methods:**

The SIOPEN Biology group analysed 99 hetMNA NBs focussing on the prognostic significance of *MYCN* ITH.

**Results:**

Patients <18 months (18 m) showed a better outcome in all stages as compared to older patients (5-year OS in localised stages: <18 m: 0.95 ± 0.04, >18 m: 0.67 ± 0.14, *p* = 0.011; metastatic: <18 m: 0.76 ± 0.15, >18 m: 0.28 ± 0.09, *p* = 0.084). The genomic 'background’, but not MNA clone sizes, correlated significantly with relapse frequency and OS. No relapses occurred in cases of only numerical chromosomal aberrations. Infiltrated bone marrows and relapse tumour cells mostly displayed no MNA. However, one stage 4s tumour with segmental chromosomal aberrations showed a homogeneous MNA in the relapse.

**Conclusions:**

This study provides a rationale for the necessary distinction between heterogeneous and homogeneous MNA. HetMNA tumours have to be evaluated individually, taking age, stage and, most importantly, genomic background into account to avoid unnecessary upgrading of risk/overtreatment, especially in infants, as well as in order to identify tumours prone to developing homogeneous MNA.

## Introduction

Homogeneous *MYCN* amplification (homMNA) was first described in neuroblastoma (NB) more than three decades ago^1,2^ and is, thus far, considered the marker of poor outcome par excellence with clinical and treatment implications in NB.^3,4^ The *MYCN* oncogene is found to be amplified in approximately 20% of all NBs and allocates patients into high-risk pretreatment groups independent of the status of other clinico-biological prognostic factors.^3,5^ HomMNA NB is frequently associated with unfavourable histology, diploidy, 1p deletion and 17q gain, whereas it is inversely associated with other genetic alterations, such as 11q aberration and *ATRX* mutations.^6–9^ Event-free survival (EFS) in these patients has improved with intensification of therapy.^10–12^

Intratumoural heterogeneous MNA (hetMNA) refers to the coexistence of MNA cells as a cluster (focus) or as single scattered cells and non-*MYCN*-amplified (nonMNA) tumour cells; in addition, tumour cells with *MYCN* gain (MNG, for definitions, see Materials and methods) can be present.^13–29^ HetMNA, thus far reported infrequently, can occur spatially within the tumour as well as between tumour and metastasis at the same time point^15,20,22,24,25,27,28^ or temporally during disease course.^14,15,17,19–21,24,26–29^

Aside from *MYCN* amplification, seven segmental chromosome aberrations (SCAs) occur repeatedly in NBs and are considered to have prognostic impact (see Materials and methods; refs. ^6,8,30^). While these NB-typical genomic findings have already been implemented into the therapeutic decision strategy (International Neuroblastoma Risk Group, INRG, European low and intermediate risk NB study, LINES; ClinicalTrials.gov identifier NCT01728155), no specific therapeutic strategies exist for patients with hetMNA NBs, due to the unclear biological and clinical impact of hetMNA. Recently, two large cohorts of hetMNA NB patients have been individually studied and compared with other genetic subgroups of NB patients.^26,28^ In both cohorts, genetic subtypes different from homMNA were frequently associated with hetMNA, e.g. 11q aberrations or whole-chromosome uniparental disomies (wcUPDs) of chromosome 11. In the Spanish cohort (*n* = 28), patients >18 months at diagnosis with an SCA profile, partly combined with SCA heterogeneity, prevailed.^26^ In the Austrian cohort (*n* = 26), age <18 months at diagnosis was clearly associated with wcUPDs (especially for chromosome 11); age >18 months, however, was associated with a multitude of SCAs and occurrence of intragenic *ATRX* deletions.^28^ The genetic findings of both studies corroborate the differences in the genetic ‘background’ between hetMNA and homMNA tumours, but the implications of MNA heterogeneity for the treatment remain to be solved.

Owing to the difficult diagnosis of hetMNA tumours, leading to undetected cases and infrequent descriptions, and their broad clinical range in terms of disease stage and outcome, the inclusion of hetMNA patients in pretreatment stratification systems has, so far, not been accomplished. In order to address the difficulties in the prognostic assessment of hetMNA tumours, the International Society of Paediatric Oncology European NB (SIOPEN) Biology Group launched a study focussing on this NB subtype. This work presents the clinical and genetic data of 99 hetMNA NB patients with the aim to elucidate the prognostic impact of MNA clones in otherwise nonMNA NBs. It will provide recommendations regarding treatment strategies for a significant proportion of hetMNA NB patients.

## Materials and methods

### Patients, centres and study protocols

Diagnostic hetMNA tumour material from 99 NB patients was collected between 1991 and 2015 at eight institutions from the following countries: Austria (26), Czech Republic (4), France (3), Israel (5), Italy (24), Norway (6), Spain (30), and United Kingdom (1). One institution also received tumour material from Poland (6) and Germany (2). Ethical approval for the diagnostic analysis was granted by local ethics commissions. Patients were staged according to the International Neuroblastoma Staging System (INSS) and treated either according to European protocols (SIOP-Europe High-Risk Neuroblastoma Study 1, SIOPEN HR-NBL1, *n* = 46; Infant Neuroblastoma European Study, INES, *n* = 7; Localised Neuroblastoma European Study Group, LNESG1/2, *n* = 9; LINES, *n* = 4) or national protocols (*n* = 16; from these patients, 8 received high-dose treatment). The exact study protocol was unknown for 10 patients, who received chemotherapy, and 5 without upfront cytotoxic therapy. For one patient, treatment was unknown. One patient died after surgery. In total, 17 patients did not receive upfront cytotoxic therapy (10 stage 1, 5 stage 2, 1 stage 4s, 1 stage unknown).

### Definitions of hetMNA and MNA clone sizes

Since techniques and terms for molecular genetics of NBs were standardised by the SIOPEN Biology group, the term 'amplification' applies to a more than four-fold increase in the *MYCN* signal number compared to reference probes on chromosome 2.^18^ Besides MNA cells, tumour cells with MNG, (signal increase ≤4-fold) can also be present. MNA heterogeneity determines the coexistence of *MYCN*-amplified and nonMNA tumour cells. It ranges from two clearly *MYCN*-amplified cells, for which artefacts have to be excluded, up to a high percentage of MNA cells occurring besides tumour cells without any supernumerary *MYCN* copies. MNA clone sizes are classified into five categories related to tumour cells without MNA: <1, 1–5, 6–10, 11–50, and >50% MNA cells. The tumours were centrally reviewed by the SIOPEN Biology group. Possible pitfalls leading to false-positive results have been recorded, categorised and carefully analysed. Among them are: contamination, cross-hybridisation, use of an inappropriate reference probe, and polyploidisation after cytotoxic treatment.

### Detection methods of hetMNA

Fluorescence in situ hybridisation (FISH) analyses for *MYCN* were performed on tumour imprints, cytospins (from disseminated tumour cells (DTCs), from the bone marrow (BM)) and/or frozen/paraffin sections in all cases as described.^18,31^ *MYCN* probes (2p24; from Kreatech Biotechnology, Amsterdam, The Netherlands; Oncor, Gaithersburg, USA and Vysis, Illinois, USA; and MetaSystems, Germany) together with internal standards such as LAF (2q11; Kreatech, Oncor, Vysis; MetaSystems, Germany), centromere-specific probes D2Z (Oncor), 2p probes (kind gift from M. Rocchi, University of Bari, Bari, Italy) or 2qter probes (Kreatech, Biotechnology) were used, with 4,6-diamidino-2-phenylindole, DAPI, as a counterstain. In addition, detection of hetMNA was also possible by multi-/pan-genomic techniques (see below) as a minor peak at the *MYCN* locus in case of sufficient tumour cell content and MNA clone sizes above the detection limit.

### Detection methods of typical and atypical SCAs and definitions

SCAs were either detected by FISH (1p36 (D1Z2)/centromere chromosome 1, Qbiogene, Illkirch, France; MLL(11q23)/SE11 and MPO(17q22) ISO17q/p53(17p53), Kreatech Biotechnology) or by multi- (multiplex ligation-dependent probe amplification (MLPA)) and pan-genomic techniques. MLPA kits and arrayCGH/SNP array platforms used were: P251/P252/P253 (MRC-Hollland, Amsterdam, The Netherlands); SurePrint G3 Human CGH Microarray Kit 4× 180K SNP array, 185,428 markers (Agilent Technologies, Santa Clara, CA, USA); Cytoscan HD, 2.67 million markers; Genechip Human Mapping Nsp Array, 262,256 markers (Affymetrix Inc., Santa Clara, CA, USA); and HumanCytoSNP-12 DNA Analysis BeadChip, 299,140 markers (Illumina, San Diego, CA, USA). Previously described protocols and INRG guidelines were followed.^6,8,32–34^ The seven SCAs designated as typical (typSCA) of NB were: losses at 1p, 3p, 4p and 11q and gains at 1q, 2p and 17q. All other segmental aberrations were designated as atypical (atypSCA).^6,30^ The term ‘numerical chromosome aberrations’ (NCA) is only used for tumours with gain of whole chromosomes, without any SCAs and without amplicons other than *MYCN*. DNA extraction and single techniques were performed as described elsewhere.^6,26,28^

### Ploidy determination and definitions

Flow cytometry (FCM) or image cytometry (ICM) were performed as described.^17,18^ Moreover, the presence or absence of numeric aberrations revealed by multi- or pan-genomic techniques was also used for ploidy allocation. In this study, the following ploidy groups were discerned: diploid, tetraploid, and aneuploid (DNA content ranging from 1.2 to 1.8) determined by FCM/ICM together with tumour cells showing aneusomies (trisomies) for at least 3 chromosomes detected by MLPA/aCGH/SNP array.^31,34^

### Assessment of bone marrow infiltration and DTC genetics

BM infiltration was diagnosed cytomorphologically and/or by flow cytometry.^35,36^ In two institutions, BM cytospin preparations were stained for GD2-positive tumour cells. If present, the coordinates of the GD2-positive cells were documented and a *MYCN*-FISH was performed and analysed after relocation of the positive cells using an automatic fluorescence microscope (Metacyte Metasystems, Germany) array.^31^

### Statistics

EFS was defined as time from diagnosis to first relapse, progression, second malignancy or death or time of last contact if no event occurred. Overall survival (OS) is defined as time from diagnosis to death or time of last contact. EFS and OS were estimated using the Kaplan–Meier method and compared with log-rank test.^1^ The cumulative incidence of relapse/progression (CIR) was estimated taking into account the competing risk of death without relapse/progression (non-relapse mortality (NRM)). CIR was compared with Grey’s test.^37^

Risk factors evaluated were age (dichotomised in patients above and below 18 months), INSS stage, SCAs and tumour cell ploidy. Two-sided *p*-values <0.05 were considered statistically significant.^37–39^

## Results

### Young age and localised tumours are common for hetMNA tumours

**Fig. 1:**
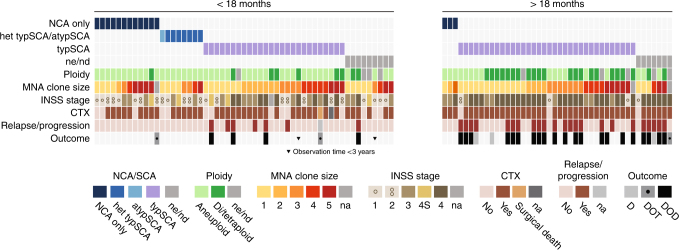
Oncoplot summarising clinical and genetic data. All hetMNA tumours for the two age groups, below (**a**) and above (**b**) 18 months of age, are shown separately and arranged according to the absence or presence of segmental chromosome aberrations, indicating also ploidy, MNA clone sizes in the analysed samples and clinical data: INSS stage, treatment, the presence of relapse or progressions, and outcome. In case of no events, observation times are at least 3 years, with the exception for two patients which are indicated. NCA numerical chromosome aberrations, het typSCA heterogeneous typical segmental aberrations, atypSCA atypical segmental aberrations, typSCA typical segmental aberrations, ne not evaluable, nd no data, *MNA* *MYCN* amplification, *INSS* International Neuroblastoma Staging System, *CTX* cytotoxic therapy, *D* dead, not disease-related, *DOD* dead of disease, *DOT* dead of therapy. Note: MNA clone size categories: 1: <1; 2: 1–5; 3: 6–10; 4: 11–50; 5: >50%

**Fig. 2:**
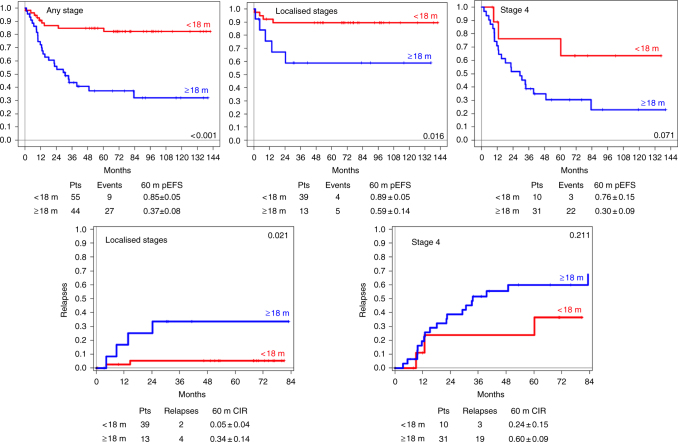

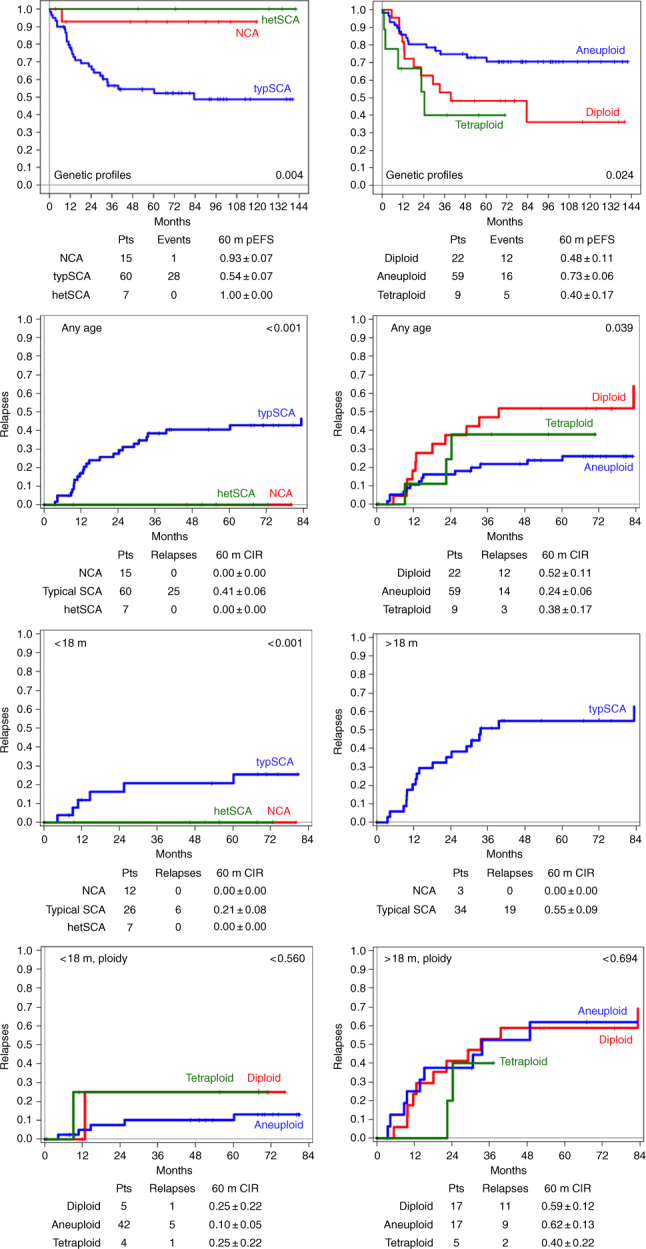
Kaplan–Meier event-free survival curves by INSS stages, age and genomic status. Sixty-month event-free survivals (EFS) for stage (any stage, localised stages and metastatic stages) according to age (first row) and cumulative incidence of relapses (CIRs) (second row); 60-month EFS according to the genomic background (third row; left: according to presence or absence of numerical and typical segmental chromosome aberrations, NCA, SCA; right: according to ploidy); CIR according to genomic background, any age (fourth row); CIR according to NCA/SCA in the two age groups (fifth row); CIR according to ploidy in the two age groups (sixth row). *pts* patients, *rel* relapse; *m* months

The age of the 99 patients at diagnosis ranged between 1 and 171 months (mean: 26.4 months; median: 14 months) with 55 patients in the younger and 44 in the older age group (see supplementary Fig. 1A). Fifty-two patients had localised stages (<18 months: 10 stage 1, 14 stage 2, 15 stage 3; >18 months: 3 stage 1, 1 stage 2, 9 stage 3). Forty-six patients suffered from disseminated disease (<12 months: 5 stage 4s: <18 months: 10 stage 4; >18 months: 31 stage 4). For one patient of the younger age group, the stage was unknown. In all, 72% (39/54) of patients in the younger age group had localised disease, as opposed to 30% (13/44) in the older age group. Localisations according to age are given in supplementary Fig. 1B. An oncoplot summary shows clinical and genomic data also according to the two age groups (Fig. 1). In Table 1, 5-year EFS and OS as well as relapses and NRM are listed for clinical and genetic parameters and their combinations. Figure 2 shows Kaplan–Meier EFS curves and CIRs by INSS stages, age and genomic status.

**Table 1:** Summary of individual clinical and genetic parameters and their combinations for the whole patient cohort

	Pts	*n*rel	5y rel/progr.	*n*NRM	5y-NRM	5y-EFS	Death	5y-OS	*p*
Age at diagnosis									
<18 m	55	7	0.12 ± 0.05	2	0.04 ± 0.03	0.85 ± 0.05	6	0.90 ± 0.04	**0.000**
>18 m	44	23	0.53 ± 0.08	4	0.09 ± 0.04	0.37 ± 0.08	25	0.36 ± 0.08	
*p*			**<0.001**		0.280	**<0.001**			
INSS stage									
Stages 1, 2	28	2	0.07 ± 0.05	1	0.04 ± 0.04	0.89 ± 0.06	2	0.93 ± 0.05	**0.000**
Stage 3	24	4	0.18 ± 0.08	2	0.08 ± 0.06	0.74 ± 0.09	4	0.83 ± 0.08	
Stage 4s	5	2	0.40 ± 0.22	0	0.00 ± 0.00	0.60 ± 0.22	1	0.75 ± 0.22	<
Stage 4	41	22	0.52 ± 0.08	3	0.07 ± 0.04	0.41 ± 0.08	24	0.39 ± 0.08	
*p*			**0.004**		0.772	**<0.001**			
Segmental chromosome aberrations									
NCA	15	0	0.00 ± 0.00	1	0.07 ± 0.07	0.93 ± 0.07	1	0.93 ± 0.07	**0.016**
het typSCA	7	0	0.00 ± 0.00	0	0.00 ± 0.00	1.00 ± 0.00	0	1.00 ± 0.00	
typSCA	60	25	0.41 ± 0.07	6	0.10 ± 0.04	0.54 ± 0.07	23	0.59 ± 0.07	
*p*			**<0.001**		0.672	**0.004**			
Tumour cell ploidy									
Aneuploid	59	14	0.24 ± 0.06	2	0.03 ± 0.02	0.73 ± 0.06	13	0.77 ± 0.06	
Diploid	22	12	0.52 ± 0.11	0	0.00	0.48 ± 0.11	11	0.47 ± 0.11	**0.030**
Tetraploid	9	3	0.38 ± 0.17	2	0.22 ± 0.14	0.40 ± 0.17	4	0.44 ± 0.21	
*p*			**0.039**		0.012	**0.024**			
Stage and age at diagnosis									
Localised <18 m	39	2	0.05 ± 0.04	2	0.05 ± 0.04	0.89 ± 0.05	2	0.95 ± 0.04	**0.011**
Localised >18 m	13	4	0.34 ± 0.14	1	0.08 ± 0.07	0.59 ± 0.14	4	0.67 ± 0.14	
*p*			**0.021**		0.719	**0.016**			
Stage 4 <18 m	10	3	0.24 ± 0.15	0	0.00	0.76 ± 0.15	3	0.76 ± 0.15	0.084
stage 4 >18 m	31	19	0.60 ± 0.09	3	0.10 ± 0.05	0.30 ± 0.09	21	0.28 ± 0.09	
*p*			0.211		**<0.001**	0.071			
Segmental chromosome aberrations and age at diagnosis									
NCA <18 m	12	0	0.00 ± 0.00	1	0.08 ± 0.08	0.92 ± 0.08	1	0.92 ± 0.08	0.493
het typSCA <18 m	7	0	0.00 ± 0.00	0	0.00 ± 0.00	1.00 ± 0.00	0	1.00 ± 0.00	
typSCA <18 m	26	6	0.21 ± 0.08	1	0.04 ± 0.04	0.75 ± 0.09	4	0.88 ± 0.07	
*p*			**<0.001**		0.326	0.166			
NCA >18 m	3	0	0.00 ± 0.00	0	0.00 ± 0.00	1.00 ± 0.00	0	1.00 ± 0.00	0.156
typSCA >18 m	34	19	0.55 ± 0.09	2	0.06 ± 0.04	0.39 ± 0.09	19	0.36 ± 0.09	
*p*			**NA**		**NA**	NA			
Tumour cell ploidy and age at diagnosis									
Aneuploid <18 m	42	5	0.10 ± 0.05	2	0.05 ± 0.03	0.85 ± 0.06	4	0.93 ± 0.04	
Diploid <18 m	5	1	0.25 ± 0.22	0	0.00 ± 0.00	0.75 ± 0.22	1	0.75 ± 0.22	0.430
Tetraploid <18 m	4	1	0.25 ± 0.22	0	0.00 ± 0.00	0.75 ± 0.22	1	0.75 ± 0.22	
*p*			0.560 (NA)		NA	0.831			
Aneuploid >18 m	17	9	0.62 ± 0.13	0	0.00	0.38 ± 0.13	9	0.34 ± 0.13	
Diploid >18 m	17	11	0.59 ± 0.12	0	0.00	0.41 ± 0.12	10	0.40 ± 0.12	0.742
Tetraploid >18 m	5	2	0.40 ± 0.22	2	0.40 ± 0.22	0.20 ± 0.18	3	0.60 ± 0.22	
*p*			0.694		NA	0.326			
Segmental chromosome aberrations, stage and age at diagnosis									
NCA, localised <18 m	11	0	0.00 ± 0.00	1	0.09 ± 0.09	0.91 ± 0.09	1	0.91 ± 0.09	0.773
het typSCA, localised <18 m	6	0	0.00 ± 0.00	0	0.00 ± 0.00	1.00 ± 0.00	0	1.00 ± 0.00	
typSCA, localised <18 m	14	2	0.14 ± 0.09	1	0.07 ± 0.07	0.79 ± 0.11	1	0.93 ± 0.07	
*p*			**<0.001**		NA	0.394			
NCA, localised >18 m	2	0	0.00 ± 0.00	0	0.00 ± 0.00	1.00 ± 0.00	0	1.00 ± 0.00	0.447
typSCA, localised >18 m	10	4	0.40 ± 0.15	1	0.10 ± 0.09	0.50 ± 0.16	4	0.60 ± 0.15	
*p*			NA		NA	NA			
typSCA, stage 4 <18 m	9	2	0.13 ± 0.12	0	0.00 ± 0.00	0.88 ± 0.12	2	0.88 ± 0.12	
*p*									
NCA, stage 4 >18 m	1	0		0			0		
typSCA, stage 4 >18 m	24	15	0.59 ± 0.10	1	0.04 ± 0.04	0.36 ± 0.10	15	0.32 ± 0.10	
*p*			NA		NA	NA			
Tumour cell ploidy, stage and age at diagnosis									
Aneuploid, localised <18 m	35	2	0.06 ± 0.04	2	0.06 ± 0.04	0.88 ± 0.06	2	0.94 ± 0.04	
Diploid, localised <18 m	2	0		0			0	1.00 ± 0.00	0.731
*p*			NA		NA	NA			
Aneuploid, localised >18 m	8	3	0.43 ± 0.19	0	0.00 ± 0.00	0.57 ± 0.19	3	0.57 ± 0.19	
Diploid, localised >18 m	2	0		0			0	1.00 ± 0.00	0.500
Tetraploid, localised >18 m	2	1	0.50 ± 0.35	1	0.50 ± 0.35	0.00 ± 0.00	1	0.50 ± 0.35	
*p*			NA		NA	NA			
Aneuploid, stage 4 <18 m	4	1	0.00 ± 0.00	0	0.00 ± 0.00	1.00 ± 0.00	1	1.00 ± 00	NA
Diploid, stage 4 <18 m	3	1	0.50 ± 0.35	0	0.00 ± 0.00	0.50 ± 0.35	1	0.50 ± 0.35	
Tetraploid, stage 4 <18 m	2	1	0.50 ± 0.35	0	0.00 ± 0.00	0.50 ± 0.35	1	0.50 ± 0.35	
*p*			NA		NA	NA			
Aneuploid, stage 4 >18 m	9	6	0.70 ± 0.16	0	0.00	0.30 ± 0.16	6	0.26 ± 0.16	0.992
Diploid, stage 4 >18 m	15	11	0.67 ± 0.12	0	0.00	0.33 ± 0.12	10	0.31 ± 0.12	
Tetraploid, stage 4 >18 m	3	1	0.33 ± 0.27	1	0.33 ± 0.27	0.33 ± 0.27	1	0.67 ± 0.27	
*p*			0.588		NA	0.867			

Statistic results for 5-year relapse frequencies, non-relapse mortality, 5-year event-free and overall survival in the individual age and INSS stage subgroups as well as the genomic subgroups (segmental chromosome aberrations and ploidy) and their combinations. Stage 4s tumours are not included because of small case numbers

Statistically significant (<0.05) p-values are indicated in bold. *atyp* atypical, *EFS* event-free survival, *pts* patients, *n* number, *het* heterogeneous, *INSS* International Neuroblastoma Staging System, *NA* not applicable, *NCA* numerical chromosome aberrations only, *NRM* non-relapse mortality, *OS* overall survival, *p* *p*-value, *progr.* progression, *rel* relapses, *SCA* segmental chromosome aberrations, *typ* typical, *y* year

### The clinical behaviour of hetMNA depends on the tumour genomic background, which is associated with age, stage and outcome

A favourable genomic tumour background was found in 23 hetMNA tumours if NCA tumours (*n* = 15), aneuploid tumours with heterogeneously present typSCA (*n* = 7) and one additional aneuploid tumour with one single atypSCA were included (excluded from the Kaplan–Meier estimations). All tumours were aneuploid (in the triploid, pentaploid, hexaploid range), except for one which was tetraploid (Fig. 1). These 'favourable background' tumours were predominantly detected in the younger age group (20/23) and mostly in localised stages (*n* = 20; 2 stage 4s, 1 stage 4). Relapses did not occur (see Table 1, Figs. 1 and 2). By contrast, an unfavourable genomic tumour background, i.e. the presence of typSCAs was found not only in the majority of hetMNA patients of the older age group (34/37 with SCA data) but also in more than half (26/46 with SCA data) of the younger age group. The typSCA tumours comprised almost all diploid/tetraploid tumours (26/27, the only exception, a tetraploid NCA tumour, is mentioned above; further four diploid tumours are without SCA information). Aneuploid tumours, by contrast, showed typSCAs in 58% (30/52).

In addition, tumours with unfavourable tumour background comprised the vast majority of disseminated stages (38/41; diploid tumours without SCA information included). In contrast to the favourable genomic background subgroup, relapses also occurred in case of localised disease (6/24, 3 patients died of disease). Altogether, an unfavourable typSCA background correlated significantly with relapse frequency and decreased OS (5-year EFS and OS: 0.54 ± 0.07 and 0.59 ± 0.07), however, with clear differences between the two age groups (Table 1, and Fig. 1). In case of a favourable NCA and heterogeneous typSCA background, 5-year EFS and OS were 0.93 ± 0.07 and 1.00 ± 0.00, respectively.

The difference in survival between the two age groups according to tumour stage irrespective of genomic background was statistically significant for localised stages (5-year EFS: <18 months: 0.89 ± 0.05; >18 months: 0.59 ± 0.14; *p* = 0.016; 5-year OS: <18 months: 0.95 + 0.04; >18 months: 0.67 ± 0.14; *p* = 0.011). For stage 4, the outcome differed as well but did not reach statistical significance, possibly due to the rather small patient number (5-year EFS: <18 months: 0.76 ± 0.15; >18 months: 0.30 ± 0.09; *p* = 0.071; 5-year OS: <18 months: 0.76 ± 0.15; >18 months: 0.28 ± 0.09; *p* = 0.084 (Table 1 and Fig. 2).

### MNA clone sizes did neither correlate with age or stage nor with the genomic background

The distribution of the different MNA clone sizes in the two age groups and the individual stages did not differ (see supplementary Fig. 2A and B). MNA clones >50% were found in localised tumours at a similar frequency as in disseminated tumours and also in tumours with a so-called favourable genomic background, i.e. aneuploid without SCA (Fig. 1). However, the investigated tumour samples are not necessarily representative of the whole tumour and overestimation or underestimation of the MNA clone(s) may occur.

### MNA clone cells do not preferentially disseminate to the bone marrow

Thirty of the 92 patients (7 BMs without information) had BM infiltration at diagnosis (6 <18 months, 24 >18 months) with known *MYCN* status for 12 patients. In four cases, the disseminated cells showed hetMNA, and in eight cases, *MYCN* was not amplified (Table 2).

**Table 2:** *MYCN* data for disseminated tumour cells in the bone marrow and tumour material from relapse or progression

Pt. no.	Age at dx in months	INSS stage at dx	MNA in BM-DTCs at dx	Relapse/progression	Time to relapse/progression in years	Analysed relapse/progression material	MNA in relapse/progression
33	12	2^a^	No	No	—	—	—
39	13	4	No	No	—	—	—
41	13	3^a^	No	No	—	—	—
58	19	4	hetMNA	Yes	1	na	—
63	23	4	hetMNA	Yes	0, 7	na	—
66	26	4	hetMNA^b^	Yes	0, 8	na	—
82	46	4	hetMNA^c^	Yes	1	Liquor	hetMNA^d^
84	46	4	No	Yes	2, 4	DTCs	No
86	50	4	No	Yes	0, 3	na	—
89	57	4	No	Yes	1, 9	na	—
90	61	4	ne	Yes	7, 10, 11	Metastases	1. rel: hetMNA; 2. rel: no MNA; 3. rel: no MNA
97	93	4	No	Yes	2, 5	DTCs	No
99	171	4	No	Yes	0, 7	Tumour	No
11	5	4s	No BM infiltration	Yes	2, 1	Tumour	MNA
48	14	3	No BM infiltration	Yes	1, 2	Tumour	No
77	41	2	No BM infiltration	Yes	0, 3	Tumour	No
78	42	4	No BM infiltration	Yes	0, 5	Lung metastasis	No

*MYCN* status for 17 patients with either tumour-infiltrated bone marrows and *MYCN* FISH investigations and/or *MYCN* FISH-investigated tumour material from relapse or progression material

*BM* bone marrow, *DTC* disseminated tumour cells, *dx* diagnosis, hetMNA heterogeneous MYCN amplification, *INSS* International Neuroblastoma Staging System, *MNA* MYCN amplification (homogeneous), *na* not available, *ne* not evaluated, *Pt. no.* patient number

^a^cytomorphologically, the bone marrows of these patients at diagnosis were free of tumour cells, however, immunofluorescence (GD2) and FISH positive

^b^majority of DTCs showed MNA

^c^5 of 12 DTCs showed MNA

^d^5 of 33 tumour cells with MNA

### Transition of hetMNA to homMNA is probably a rare event in patients >18 months but may occur occasionally in patients <18 months

For seven patients of the older age group, the *MYCN* status in relapse tumour material was analysed (Table 2). In five of them, no MNA was detectable. In one patient, hetMNA persisted with 5 MNA cells besides 28 nonMNA tumour cells (verified by 2p gain). In a further patient, who experienced three relapses, hetMNA was found in the first relapse but the following two showed no MNA. In the younger patient group, a stage 4s patient with typSCAs showed a homMNA at relapse, a further stage 2 patient had a relapse without MNA.

### Upgrading of risk based on heterogeneous *MYCN* status leading to cytotoxic treatment with or without high-dose chemotherapy with stem cell reinfusion

Patients with a hetMNA diagnosis were either categorised as having no MNA or homMNA. Thus the question of upgrading of risk of hetMNA patients at diagnosis to conventional or high-dose chemotherapy with stem cell rescue concerned 60 patients who had localised disease (any age), stage 4s or stage 4 diseases (<12 months of age). Seventeen patients did not receive upfront cytotoxic treatment (age: 14 <18 months, 3 >18 months; stage: 10 stage 1, 5 stage 2, 1 stage 4s and 1 unknown stage). In this patient subgroup, three relapses occurred: a stage 2 patient <18 months with a local relapse, a further stage 2 patient >18 months at diagnosis without MNA in the relapse, and a stage 4s patient with a homMNA relapse. All three patients had typSCA tumours. The two latter patients died of disease. Fifteen patients received conventional cytotoxic therapy (3 stage 1, 4 stage 2, 6 stage 3, 1 stage 4s, 1 stage 4). One stage 3 patient >18 months at diagnosis with a typSCA tumour relapsed and died of disease. Twenty four patients (5 stage 2, 15 stage 3, 2 stage 4s, 2 stage 4 <12 months) received high-dose cytotoxic treatment. In this patient subgroup, two relapses occurred, one patient died of disease, one due to toxicity and one further death was not disease related. Four patients got upfront cytotoxic treatment; however, the information whether it was conventional or high-dose cytotoxic treatment with stem cell reinfusion was lacking. The data are shown in Table 3. In summary, an upgrading of risk based on a heterogeneous *MYCN* status occurred in 31 patients (7 stage 1 and 2 patients received conventional cytotoxic therapy, 24 high-dose chemotherapy with stem cell reinfusion). Twenty five patients were not upgraded despite of the heterogeneous *MYCN* amplification status.

**Table 3:** Therapeutic decisions for hetMNA patients with localised, INSS 4s and 4 (<12 months) tumour stages

	No CTX	Conventional dose CTX	High-dose CTX with stem cell reinfusion
Stage 1	10^1^	3^2,3^	—
Relapse	0	0	—
DOD/DOT	0	0	—
Stage 2	5^1^	4^2^	5^2^
Relapse	2	0	0
DOD/DOT	1 DOD^ugb,^^a^	0	1 DOT^fgb^
Stage 3	—	6^1^	15^2^
Relapse	—	2	2
DOD/DOT/D	—	1 DOD^ugb^	1 DOD^ugb^, 1 D
Stage 4s	1^1^	1^1^	2^2^
Relapse	1	0	0
DOD/DOT	1 DOD^ugb,b^	0	0
Stage 4 <12 m	—	1^1^	2^2^
Relapse	—	0	0
DOD/DOT	—	0	0
Stage unknown	1^1^	—	—
Relapse	0	—	—
DOD/DOT	0	—	—
Total	17	15	24
Relapse	3	2	2
DOD/DOT	2 DOD	1 DOD	1 DOD, 1 DOT, 1 D

Fifty six patients with localised stages, stage 4s or stage 4 (<12 months at diagnosis) tumours are categorised according to therapeutic decisions into three treatment groups: no cytotoxic therapy (CTX), conventional CTX, or high-dose CTX for high-risk neuroblastoma (NB) patients

Four patients received cytotoxic treatment; however, information on the exact regimen was lacking: 1 stage 2, 2 stage 3 (no relapses), 1 stage 4s (relapse, patient alive). All three patients with localised stages who died of disease were >18 months of age at diagnosis and had an unfavourable genomic background tumour. The hetMNA patient who died OWING to toxicity was <18 months at diagnosis and had a tumour with only numeric chromosomal aberrations

*CTX* chemotherapy, *DOD* dead of disease, *DOT* death due to toxicity, *D* death not tumour-related, *HR* high risk, *INSS* International Neuroblastoma Staging System, *fgb* favourable genomic background tumour, *ugb* unfavourable genomic background tumour

Upgrading of risk according to the *MYCN* status (hetMNA): ^1^no upgrading, ^2^upgrading, ^3^two patients were treated according to LINES G9 (low and intermediate risk neuroblastoma treatment group 9 for stage 1 neuroblastomas with *MYCN* amplification)

^a^No MNA in the relapse material

^b^homMNA in the relapse material

## Discussion

The discovery of the high clinical relevance of ITH, which includes gene amplifications, gene mutations, epigenetic changes and also segmental gains and losses (for a summary see refs. ^40–42^) attracts increasing attention in adult cancer as well as paediatric cancer research.^43,44^ Owing to its impact on relapse and/or therapy resistance, ITH has become an attractive and important topic in NB.^45,46^ Heterogeneity for the *MYCN* status as found in NB has, to the best of the authors’ knowledge, not been described for other paediatric tumour entities known for the occurrence of MNA, including medulloblastoma, rhabdomyosarcoma, nephroblastoma or retinoblastoma.^47–50^

ITH was first described for NB in 1996, followed by only infrequent reports showing patient outcome ranging from favourable to unfavourable (see Table 4). Owing to the lack of larger studies, sampling error and/or the detection limit of multi-/pan-genomic techniques analysing bulk tumour DNA,^26,28^ an estimated number of undetected cases can be assumed. Sampling error can lead to both false-negative cases (reported as nonMNA) as well as false-positive cases (reported as homMNA). Thus hetMNA detection may vary substantially depending on the amount of obtained tumour material (tumour resection, surgical biopsy, needle biopsy), on sampling and on the techniques applied. In previous publications, the authors reported a hetMNA frequency of 9.7–11.8%.^26,28^

**Table 4:** Summary of studies that reported on heterogeneous *MYCN* amplification in NB

Study	*n* cases (%)	Technique	Sample (*n* cases)	HetMNA		Age >/<18 months (*n*)	Stage (*n*)	Outcome (*n*)
				Patterns	Features (*MYCN* copies)			
Squire et al., 1996	5/29 (17.2)	FISH, SB, PCR	PT	Intratissue	2/5 with small number of MNA cells (3–10)	NR	NR	NR
Lorenzana et al., 1997	1	FISH, SB	PT & CTX	Intratissue	GNB: MNA u-nbs (15–20), nonMNA d-nbs	>	3	DOD
Ambros et al., 2001	3/300 (1)	FISH	PT	Intratissue	Focal MNA (10–50)	NR	1 (2), 4s (1)	NR
Kerbl et al, 2002	1	FISH	PT	Intratissue	Focal MNA (>15)	<	NR	ADF
Noguera et al., 2003	1	FISH	PT & R	Temporal	PT: MNG, R: homMNA	<	4s	DOD
Valent et al., 2004	4/200 (2)	FISH, Q-PCR	PT (4), BM (1)	Intratissue & spatial	MNA and MNG cells; PT: homMNA, BM: MNG	NR	4s (2), 3 (1), 4 (1)	ADF (1) AWT (2) DOT (1)
Spitz et al., 2004	4/659 (<1)	FISH	PT (2), BM (1), CTX (1)	Intratissue, spatial & temporal	Focal and scattered MNA; PT/BM: hetMNA, R: homMNA; PT: nonMNA, BM: MNA	NR	NR	NR
Thorner et al., 2006	4/41 (9.7)	CISH, FISH, SB, PCR	PT	Intratissue	≥50% *MYCN* copy number difference from cell to cell	NR	NR	NR
Sano et al., 2007	1	FISH	PT	Intratissue & spatial	Composite NB: noMNA, MNA; Lymph nodes: homMNA	<	3	AWT
Cañete et al, 2009	5/46 (10.9)	FISH	PT	Intratissue	SIOPEN cohort	<	NR^a^	NR
Theissen et al., 2009	20/1341 (1.5)	FISH	PT (15), PT & BM (3), PT & R (2)	Intratissue, spatial & temporal	Focal & scattered MNA; PT: hetMNA, R: nonMNA/hetMNA/homMNA; PT: nonMNA/homMNA, R:hetMNA/homMNA; PT: nonMNA, BM: nonMNA/hetMNA, R: hetMNA/homMNA	< (9) & > (11)	(2), 4s (3), 3 (6), 4 (9)	ADF (7) Prog. (4) DOD (9)
Bishop et al., 2014	1	FISH	PT	Intratissue, spatial	Focal MNA; adrenal mass: hetMNA, Liver: homMNA	<	4s	ADF
Berbegall et al., 2016	3	FISH, aSNP	PT	Intratissue, spatial & temporal	Iliac crests: nonMNA and MNA; BM: nonMNA, metastases: hetMNA	>	4	Prog.
Marrano et al., 2017	8/30 (26.7)	qPCR, FISH, CISH	PT/CTX (4), PT & R (1), M (1), CTX & R/M (2)	Intratissue, spatial & temporal	Changes in the *MYCN* copy number (reduction or increase)	< (2) & > (5)	(6)	DOD (6), A (2)

Note: studies of recently published patient cohorts with hetMNA tumours corresponding to refs. ^25,27^ are not included

*n* number, *FISH* fluorescence in situ hybridisation, *PCR* polymerase chain reaction, *qPCR* quantitative PCR, *SB* Southern blot, *aSNP* SNP array, *ADF* alive disease free, *AWT* alive with treatment, *A alive, CISH* chromosome in situ hybridisation, *CTX* post-chemotherapy biopsy, *d-nbs* differentiated neuroblasts, *DOD* dead of disease, *GNB* ganglioNB, MNA MYCN amplification, *homMNA* homogeneous MNA, *L* localised, *MNG* *MYCN* gain, *pd-nbs* poorly differentiated neuroblasts, *NR* not reported, *Prog.* progression, *PT* primary tumour, biopsy, *M* metastasis biopsy at diagnosis, *u-nbs* undifferentiated neuroblasts

^a^other than stage 1

With regard to the tumour genomic background of hetMNA NBs, Berbegall et al. highlighted the genomic instability associated with hetMNA tumours, including 11q deletions, predominance of advanced tumours and the need for multiple sampling.^26^ Bogen et al. stressed the possible significance of age and the genomic background for the tumours’ aggressiveness.^26,28^ However, insufficient knowledge of the impact of an MNA clone on the tumour biology has hampered biology-based treatment decision making and patients are continuously allocated to either the nonMNA or the homMNA pretreatment risk groups.

The present study reveals clear differences between hetMNA and homMNA NBs. They concern a marked age and stage dichotomy in hetMNA, differences in the genomic tumour background, including ploidy and SCAs, and in the outcome. While the majority of patients with homMNA tumours are usually >18 months of age and mostly have high-stage tumours irrespective of age, this is not the case for patients with hetMNA NBs.^6,28,51^ In this study, we show over half of the patients to be found in the younger age group and a high frequency of localised stages (54%, any age). Moreover, in contrast to homMNA, the genetic tumour background in hetMNA was more often a favourable NCA background (2 and 4% in the case of homMNA versus 18% for hetMNA; CCRI and INCLIVA databases, respectively, unpublished). In the older age group, many tumours show an SCA number often exceeding the numbers found in homMNA tumours, most likely because of the observed genetic instability in hetMNA tumours.^26,28,51^ Altogether, aberrations common in hetMNA tumours (e.g. wcUPD11 or 11q loss) are only very rarely observed in homMNA tumours^9^ or not encountered at all. The latter is the case for intragenic deletions of the *ATRX* gene.^28^ Also the outcome of hetMNA patients with localised disease differed as compared to homMNA patient: data from the LNESG1 cohort showed that five of the seven stage 1 homMNA tumour patients without upfront cytotoxic treatment experienced a relapse and four patients died of disease.^52^ By contrast, none of the 13 stage 1 hetMNA patients showed a relapse (10 patients without upfront cytotoxic treatment, 3 patients with conventional chemotherapy). For stages 2 and 3 patients with homMNA treated in the SIOPEN HR-NBL1 study (29 stage 2, 160 stage 3 patients), the 5-year EFS was 85%+7 and 63%+4, respectively (personal communication U. Pötschger, R. Ladenstein). In the hetMNA cohort, the 5-year EFS for stage 2 was 85%+9 and for stage 3 patients 74%+9, although 15 of the 35 patients received either no upfront chemotherapy or only conventional chemotherapy (for 2 further patients therapy is unclear, 1 patient died owing to surgical complications).

In the younger age group, MNA clones often develop in otherwise favourably behaving tumours with MNA cells possibly still lacking full malignancy at the time of diagnosis. This assumption is supported by the fact that none of the 20 hetMNA tumours without SCAs or with only heterogeneous or atypical SCAs led to a relapse, irrespective of partly large MNA clone sizes, together with the fact that tumour cells in the BM did not show MNA. Five of these patients (4 stage 1, 1 stage 2) did not receive cytotoxic treatment. In addition, even in case of an unfavourable genetic background (typSCA and/or diploidy/tetraploidy), survival was significantly superior if compared to the older age group irrespective of tumour stage. Although the better survival of younger patients is unexplained so far, it is in line with data on unresectable nonMNA NBs^30^ as well as with LNESG data (in submission). However, although rare, the existence of aneuploid homMNA NBs with aberrations similar to aneuploid hetMNA tumours (11q deletion, wcUPDs)^26,28^ indicates that clonal expansion with outgrowth of MNA cells can occur. The only transition (one out of nine) from hetMNA to homMNA occurred in a stage 4s patient encountered in this study with an aneuploid typSCA tumour including 1p deletion and an unfavourable histology. This patient was a non-responder to the upfront chemotherapy and showed homMNA in a relapse after 2.1 years.

In most tumours of the older patient group, by contrast, MNA clones developed against a multiple SCA background,^26,28^ thus making these tumours high-risk tumours. Neither the BM DTCs at diagnosis nor the relapse material showed a progression to homMNA, but either no MNA or, less often, the coexistence of the amplified and the non-amplified clones were observed. Interestingly, one patient with three relapses showed hetMNA in the first but no MNA in the following two relapses. These observations, together with a genomic tumour background clearly different from most homMNA NBs,^26,28^ indicate an absence of selective advantage for the MNA clones in most of the advanced-stage NBs in older patients.

The clinically most relevant and so far unanswered question concerns the consequences for treatment in case of hetMNA NBs, especially whether or not high-dose cytotoxic therapy is needed in low-stage hetMNA NBs. For other cancers, there is evidence that—based on heterogeneous genetic changes with already established prognostic influence—special treatment strategies might be proposed.^40,41,53^ In breast cancer, the presence of between 5 and 50% of cells with HER2/CEP17 ratios of >2.20 has been suggested as het*HER2* amplification because of the observed EFS decrease.^54^ However, recent studies have shown some contradictory results concerning treatment responses in heterogeneously amplified *HER2* breast cancer.^55^

Regarding hetMNA NBs, this study provides information on the differences between hetMNA and homMNA tumours and shows the necessity to clearly distinguish them. In the younger age group, the discrimination is crucial to avoid upgrading to high risk and overtreatment, especially in localised stages and favourable genomic backgrounds. Of 5 stage 2 patients (all <18 months at diagnosis) upgraded to high-risk treatment, 4 had aneuploid, favourable background tumours and 1 of them died owing to toxicity of therapy. Four stage 2 patients (all <18 months at diagnosis) treated with conventional chemotherapy showed no relapses and no deaths. Finally, five further stage 2 patients received no upfront cytotoxic treatment. One of them, a 41-month-old patient (the only patient >18 months) with an unfavourable background tumour, died of disease, however, without MNA in the relapse. On the other hand, however, as already mentioned above, it has to be borne in mind that, in the younger age group, hetMNA tumours can in fact give rise to the rather rare aneuploid homMNA tumours, as was observed for one stage 4s patient. In the older age group, by contrast, not only disseminated but also localised hetMNA tumours are usually highly aggressive tumours due to the presence of frequently numerous SCAs. Biologically, these tumours most likely correspond to nonMNA tumours and not to homMNA tumours. This assumption is based on specific SCAs present in hetMNA but not in homMNA tumours^26,28^ and the presence of *ATRX* intragenic deletions in hetMNA tumours, not so far detected, in homMNA tumours^28^ and is further supported by the lack of homMNA relapses in patients >18 months at diagnosis (shown in Table 2). The biological assignment of hetMNA tumours with this kind of unfavourable genomic background to unfavourable nonMNA tumours could become essential as soon as specific and different treatment modalities for homMNA and nonMNA NB patients will be available.

The first large study of hetMNA NB performed by the SIOPEN Biology Group provides essential information on the biological/clinical behaviour of hetMNA NBs—which should be categorised as a third genetic *MYCN*-based subgroup besides homMNA and nonMNA—and points to the frequent genomic and prognostic differences between the two age groups. It also challenges. The habit of risk upgrading of hetMNA tumour patients, concerns especially the age group below 18 months and localised stages in which hetMNA should not be equated with homMNA with regard to therapeutic decisions. Based on the data presented here, we suggest launching a trial for a selected patient subgroup with resectable hetMNA tumours (sampled according to INRG guidelines^6^) to evaluate a watch-and-wait approach after surgery with close follow-up examinations. Summarising, hetMNA must not be regarded as an isolated fact but should be assessed paying special attention to the genomic tumour background in combination with the clinical pattern including age and stage.

## Electronic supplementary material

Suppl Fig, 1 and 2

Supplementary Figure Legends
