# Supplementary figures and images for: Heterogeneous MYCN amplification in neuroblastoma: a SIOP Europe Neuroblastoma Study
Source: Br J Cancer. 2018 May 14;118(11):1502–12. doi: 10.1038/s41416-018-0098-6 (PMC5988829; doi:10.1038/s41416-018-0098-6)

Supplementary Figure 1

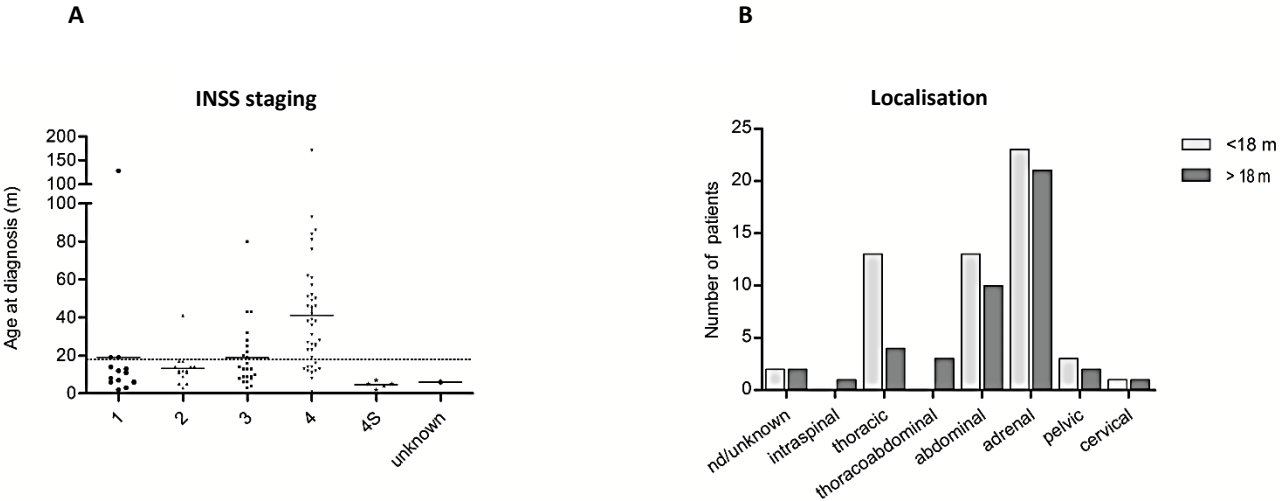

Supplementary Figure 2

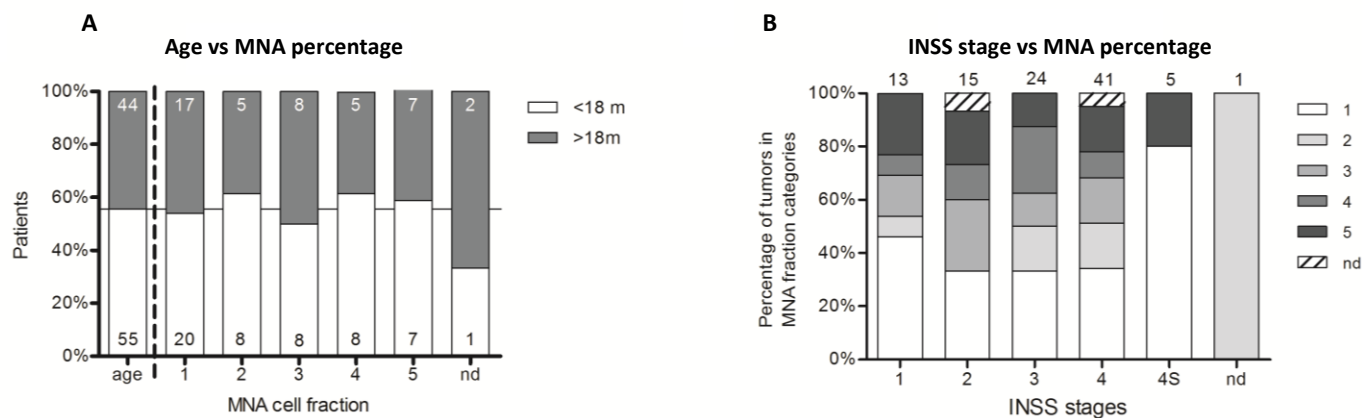

**C**

[illegible]

Supplement: Supplementary file 1 — Suppl Fig, 1 and 2 [file 41416_2018_98_MOESM1_ESM.pdf]
