# Supplementary material for: Heterogeneous MYCN amplification in neuroblastoma: a SIOP Europe Neuroblastoma Study
Source: Br J Cancer. 2018 May 14;118(11):1502–12. doi: 10.1038/s41416-018-0098-6 (PMC5988829; doi:10.1038/s41416-018-0098-6)
Supplement: Supplementary file 2 — Supplementary Figure Legends [file 41416_2018_98_MOESM2_ESM.docx]

**Supplementary Figure Legends**

**Supplementary Figure 1.** Representation of stages and tumour localisation according to the age of the patients at diagnosis. (A) Graph depicting INSS stages according to age in months. Dashed line marks the 18 months cut-off and short continuous lines depict the mean values. (B) Bar diagram showing localisations of primary tumours according to younger and older age groups.

Abbreviation: INSS = International Neuroblastoma Staging System; nd = no data

**Supplementary Figure 2**. Distribution of the MNA clone sizes according to age groups and INSS stages. (A) The bar diagram on the left side shows the distribution (in per cent) of the single MNA cell fraction categories 1 to 5 (MYCN clone sizes, for definition see M&M) in the two age groups. Numbers within the columns indicate patient counts; the diagram on the right side (B) depicts the percentages of tumours according to MNA cell fraction categories in the single INSS stages. (C) The table indicates numbers and percentages of the single MYCN clone size classes in the individual stages.

Abbreviation: INSS = International Neuroblastoma Staging System; MNA = MYCN amplification; nd = no data.
